# Supplementary material for: Development of a revised Jalowiec Coping Scale for use by emergency clinicians: a cross-sectional scale development study
Source: BMJ Open. 2019 Dec 2;9(12):e033053. doi: 10.1136/bmjopen-2019-033053 (PMC7003388; doi:10.1136/bmjopen-2019-033053)
Supplement: Supplementary data [file bmjopen-2019-033053supp001.pdf]

Supplementary Table 1. Goodness of fit for models comparing job role and country

Fixed parameters	χ^2 (df)	CFI	TLI	RMSEA	SRMR
Job Role					
Factor structure (configural model)	636 (264)	0.93	0.91	0.06	0.08
Threshold	700 (297)	0.92	0.92	0.06	0.08
Loadings	725 (312)	0.92	0.92	0.06	0.08
Country					
Factor structure (configural model)	586 (264)	0.94	0.93	0.05	0.08
Threshold	833 (297)	0.90	0.89	0.06	0.08
Loadings	927 (312)	0.88	0.89	0.07	0.08

Supplementary Table 2. Standardized factor loadings for factor structure outlined in confirmatory factor analysis for nurses and physicians

Factors and Items†	Factor Loading Nurses			Factor Loadings Physicians		
	NE	PE	PF	NE	PE	PF
Negative Emotion-Focussed Coping						
3. Used smoking or medication for stress relief	**0.50**			**0.57**		
9. Pessimistic thinking	**0.48**			**0.38**		
22. Spent time alone	**0.60**			**0.61**		
34. Drank	**0.48**			**0.36**		
46. Risky behaviour	**0.58**			**0.56**		
48. Ignored problem	**0.58**			**0.65**		
51. Self-blame for problem	**0.59**			**0.58**		
53. Took stress-reducing medications	**0.58**			**0.52**		
56. Physical distancing	**0.76**			**0.82**		
58. Wishful thinking	**0.55**			**0.55**		
Positive Emotion-focussed Coping						
37. Hardiness attitude		**0.52**			**0.51**	
39. Used humour		**0.52**			**0.55**	
50. Optimistic thinking		**0.85**			**0.71**	
54. Refocus on good side		**0.77**			**0.82**	
Problem-focussed coping						

15. Discussed problem with professional	**0.47**	**0.48**
27. Information seeking	**0.58**	**0.61**
42. Discussed problem with someone who has experienced the situation	**0.57**	**0.59**
45. Learned new skills	**0.63**	**0.55**

Supplementary Table 3. Standardized factor loadings for factor structure outlined in confirmatory factor analysis for Australia and Sweden

Factors and Items†	Factor Loading Sweden			Factor Loadings Australia		
	NE	PE	PF	NE	PE	PF
Negative Emotion-Focussed Coping						
3. Used smoking or medication for stress relief	**0.62**			**0.44**		
9. Pessimistic thinking	**0.45**			**0.46**		
22. Spent time alone	**0.54**			**0.60**		
34. Drank	**0.47**			**0.39**		
46. Risky behaviour	**0.55**			**0.57**		
48. Ignored problem	**0.42**			**0.65**		
51. Self-blame for problem	**0.53**			**0.60**		
53. Took stress-reducing medications	**0.67**			**0.54**		
56. Physical distancing	**0.63**			**0.77**		
58. Wishful thinking	**0.53**			**0.63**		
Positive Emotion-focussed Coping						
37. Hardiness attitude		**0.48**			**0.53**	
39. Used humour		**0.56**			**0.62**	
50. Optimistic thinking		**0.91**			**0.76**	
54. Refocus on good side		**0.78**			**0.77**	
Problem-focussed coping						

15. Discussed problem with professional	**0.64**	**0.43**
27. Information seeking	**0.48**	**0.62**
42. Discussed problem with someone who has experienced the situation	**0.81**	**0.54**
45. Learned new skills	**0.58**	**0.60**
