# Supplementary material for: Development of a revised Jalowiec Coping Scale for use by emergency clinicians: a cross-sectional scale development study
Source: BMJ Open. 2019 Dec 2;9(12):e033053. doi: 10.1136/bmjopen-2019-033053 (PMC7003388; doi:10.1136/bmjopen-2019-033053)
Supplement: Author's manuscript [file bmjopen-2019-033053.draft_revisions.pdf]

BMJ Open is committed to open peer review. As part of this commitment we make the peer review history of every article we publish publicly available.

When an article is published we post the peer reviewers' comments and the authors' responses online. We also post the versions of the paper that were used during peer review. These are the versions that the peer review comments apply to.

The versions of the paper that follow are the versions that were submitted during the peer review process. They are not the versions of record or the final published versions. They should not be cited or distributed as the published version of this manuscript.

BMJ Open is an open access journal and the full, final, typeset and author-corrected version of record of the manuscript is available on our site with no access controls, subscription charges or pay-per-view fees (http://bmjopen.bmj.com).

If you have any questions on BMJ Open's open peer review process please email info.bmjopen@bmj.com

BMJ Open

Development of a revised Jalowiec coping scale for use by emergency clinicians: A scale development study

Journal:	*BMJ Open*
Manuscript ID	bmjopen-2019-033053
Article Type:	Research
Date Submitted by the Author:	19-Jul-2019

Complete List of Authors:	Greenslade, Jaimi; Queensland University of Technology Faculty of Health, Institute of Health and Biomedical Innovation, School of Public Health annd Social Work; Royal Brisbane and Women's Hospital, Emergency and Trauma Centre<br>Wallis, Marianne; University of the Sunshine Coast, School of Nursing & Midwifery<br>Johnston, Amy; Princess Alexandra Hospital Health Service District, Department of Emergency Medicine; University of Queensland - Saint Lucia Campus, School of Nursing, Midwifery, and Social Work<br>Carlström, ED ; Goteborgs Universitet,<br>Wilhelms, Daniel; Linköping University, Department of Health and Medical Sciences,; Local Health Care Services in Central Östergötland, Department of Emergency Medicine<br>Thom, Ogilvie; Sunshine Coast Hospital and Health Service, Department of Emergency Medicine<br>Abraham, Louisa; Royal Brisbane and Women's Hospital,<br>Crilly, Julia; Griffith University, Menzies Health Institute; Goldcoast Health Service District, Department of Emergency Medicine

Keywords:	ACCIDENT & EMERGENCY MEDICINE, MENTAL HEALTH, Human resource management < HEALTH SERVICES ADMINISTRATION & MANAGEMENT

SCHOLARONE™
Manuscripts

DEVELOPMENT OF A REVISED JALOWIEC COPING SCALE FOR USE BY EMERGENCY CLINICIANS: A SCALE DEVELOPMENT STUDY

AUTHORS

Associate Professor Jaimi H GREENSLADE, B.Psych (Hons), M.Sci (Stats), PhD

Emergency and Trauma Centre, Royal Brisbane and Women's Hospital, Herston, QLD, 4029, Australia

Australian Centre for Health Services Innovation, School of Public Health and Social Work, Institute of Health and Biomedical Innovation, Queensland University of Technology, Kelvin Grove, QLD, 4059, Australia

Professor Marianne WALLIS, RN, BSc (Hons), PhD

Sunshine Coast Health Institute, University of the Sunshine Coast, Sippy Downs, QLD, 4558, Australia

Menzies Health Institute Queensland, Griffith University, QLD, 4222, Australia

Dr Amy N.B. JOHNSTON BN, BSc (Hons), MEd, PhD

Department of Emergency Medicine, Princess Alexandra Hospital, QLD, 4102, Australia

School of Nursing, Midwifery, and Social Work, The University of Queensland, QLD, 4072, Australia

Professor Eric CARLSTRÖM, RN, PhD

Health and crisis management and policy, Sahlgrenska Academy, University of Gothenburg Gothenburg, Sweden

University College of Southeast Norway, Vestfold, Norway

Associate Professor Daniel B WILHELMS, MD, PhD

Department of Health and Medical Sciences, Linköping University, Linköping, Sweden

Department of Emergency Medicine, Local Health Care Services in Central Östergötland, Region Östergötland,Linköping, Sweden

Dr Ogilvie THOM, MBBS, FACEM

For peer review only - http://bmjopen.bmj.com/site/about/guidelines.xhtml

Department of Emergency Medicine, Sunshine Coast Hospital and Health Service, Queensland, 4560, Australia.

Dr Louisa ABRAHAM, MBChB, BSc (Hons)
Emergency and Trauma Centre, Royal Brisbane and Women's Hospital, Herston, QLD, 4029, Australia

Professor Julia CRILLY BN, MN (Hons), PhD
Department of Emergency Medicine, Gold Coast Health, QLD, 4215, Australia
Menzies Health Institute Queensland, Griffith University, QLD, 4222, Australia

On behalf of the WES investigators
Hui (Grace) XU, MNP
Department of Emergency Medicine, Logan Hospital

Elizabeth ELDER, MN
School of Nursing and Midwifery, Griffith University

James HUGHES, PhD
Emergency and Trauma Centre, Royal Brisbane and Women's Hospital
School of Nursing, Queensland University of Technology

Monica A MAGNUSSON, MScN
Emergency Department, Linköping University Hospital, Linköping

CORRESPONDING AUTHOR
Associate Professor Jaimi H GREENSLADE
Institute of Health and Biomedical Innovation, School of Public Health and Social Work
Queensland University of Technology
Musk Avenue
KELVIN GROVE, QLD, 4059, Australia
j.greenslade@qut.edu.au
Ph: +61 7 3646 6262

For peer review only - http://bmjopen.bmj.com/site/about/guidelines.xhtml

WORD COUNT: 3180

KEY WORDS: Coping; Emergency Departments; Scale Development; Stress

ABSTRACT

Objectives: The aim of this study was to develop and validate a scale to measure the coping strategies used by emergency staff in response to workplace stress. To achieve this aim, we developed a refined Jalowiec Coping Scale, termed the Jalowiec coping scale – Emergency Department (JCS-ED) and validated this scale on a sample of emergency clinicians.

Design: A cross-sectional survey incorporating the Jalowiec coping scale was administered between July 2016 and June 2017. The JCS-ED was developed in three stages 1) item reduction through content matter experts, 2) exploratory factor analysis for further item reduction and to identify the factor structure of the revised scale, and 3) confirmatory factor analyses to confirm the factors identified within the exploratory factor analysis.

Setting: Six Emergency Departments (EDs) in Australia and four in Sweden.

Participants: Participants were eligible for inclusion if they worked full-time or part-time as medical or nursing staff in the study EDs.

Results: A total of 875 ED staff completed the survey (response rate 51%). The content matter experts reduced the 60-item scale to 32 items. Exploratory factor analyses then further reduced the scale to 18 items assessing three categories of coping; problem-focussed coping, positive emotion-focussed coping, and negative emotion-focussed coping. Confirmatory factor analysis supported this 3-factor structure. Negative coping strategies were associated with poor perceptions of the work environment and higher ratings of stress.

Conclusions: The JCS-ED assesses maladaptive coping strategies along with problem- and emotion-focussed coping styles. It is a short instrument that is likely to be useful in measuring the types of coping strategies employed by staff. This will aid in the development and evaluation of evidence-based interventions to promote positive coping while reducing the use of maladaptive coping strategies.

For peer review only

For peer review only - http://bmjopen.bmj.com/site/about/guidelines.xhtml

STRENGTHS AND LIMITATIONS OF THE STUDY

- This study utilizes a large cohort of nurses and physicians from 10 Emergency Departments in two countries.
- This coping scale has been validated for use in the emergency department and may also be relevant to the broader range of staff employed in an acute care setting.
- The JCS-ED is practical as it is short enough to encourage inclusion in future surveys exploring ED staff perceptions.
- No longitudinal data were available, meaning that test-retest reliability was unable to be determined
- The response rate was reasonable for a survey design. However, it is unknown whether there was non-response bias.

For peer review only

For peer review only - http://bmjopen.bmj.com/site/about/guidelines.xhtml

INTRODUCTION

The Emergency Department (ED) is a dynamic and demanding environment. Positive factors such as a supportive team environment, the development of high-level clinical skills, and the challenges around dealing with varied patient groups attract clinical staff to this setting [1]. However, high workload, inadequate staffing, workplace violence, and dealing with critically ill patients can place strain on employees [2]. Indeed, high levels of psychological distress [3] and staff turnover [4] are evident amongst ED staff. The coping strategies employed by clinicians may be one critical component in distinguishing these contrasting views of the ED as a stressful versus positive environment.

Coping is defined as the thoughts and behaviours used to manage the demands of situations that are appraised as stressful [5]. The transactional model of stress posits that the relationship between stressors and strain is mediated by coping. That is, effective coping reduces experience of strain [6]. Many different coping strategies can be employed, and these depend on both the environment and on personal disposition [7]. Such coping strategies either address the problem causing distress (problem-focussed coping), or ameliorate the negative emotions associated with the stressor (emotion-focussed coping) [7]. Some coping strategies may be positive and functional, while others can have negative consequences [7]. Previous studies have suggested that ED staff using problem-focussed coping have lower levels of burnout [8] and better psychological health [3] than those using emotion-oriented coping or maladaptive coping strategies. Identifying the functional and maladaptive coping strategies that are used within the ED is important as these are amenable to intervention, with therapies such as cognitive behaviour therapy being shown to successfully increase the use of beneficial strategies [7].

One major challenge in identifying and modifying coping strategies within the ED is that the objective measurement of coping has been difficult. Existing scales are lengthy [7], or have displayed poor psychometric properties [9]. The Jalowiec Coping Scale (JCS) is a useful coping scale that was developed to measure the process of coping in a healthcare setting. This scale incorporates 60 items distributed across 8 dimensions and has been translated into more than 20 languages [10]. The JCS was originally developed to measure problem- and emotion-focused coping styles, but the final version identifies 8 different dimensions of coping. The JCS

For peer review only - http://bmjopen.bmj.com/site/about/guidelines.xhtml

has been used nationally and internationally with both patients and employees in the healthcare setting [11]. While this scale has been extensively utilized, studies assessing its psychometric properties have not supported the 8-item structure [12]. Further, a 60-item scale is arguably too long for busy ED staff.

This study had two major aims. The first was to develop a coping scale for use by ED staff. This instrument should incorporate items to identify beneficial and maladaptive problem- and emotion-focused coping strategies while also being short enough for easy completion in a busy work environment. To achieve this aim, we modified the JCS coping scale for the emergency department setting. The second goal was to use this modified JCS, termed JCS-ED, to describe the coping strategies employed by ED staff across two countries; Australia and Sweden.

METHODS

Participants and Design

All full-time and part-time medical and nursing staff employed in the study EDs were eligible for inclusion in this study. A cross-sectional survey was administered between July 2016 and June 2017 depending on logistics at each site. Surveys were distributed to 1709 staff, with 876 returned. One survey was excluded due to extensive missing data, making the final sample size 875 (51% response rate). Staff were invited to participate via email and in ward-based information sessions. Surveys were returned to locked boxes within each hospital ED, or via stamped self-addressed envelopes. A reminder email was sent out two-weeks after survey distribution.

Instrument

The JCS was used to assess the coping strategies employed by ED clinicians. The JCS incorporates 60-items that were developed to measure eight different coping dimensions. These include facing up to the problem (confrontive coping), avoiding the problem (evasive coping), positive thinking (optimistic coping,) pessimistic thinking (fatalistic coping), releasing emotions (emotive coping), making yourself feel better (palliative coping), using support systems (supportive coping), and depending on yourself (self-reliant coping). Respondents report how often they have used each coping method, with scores for each item ranging from 0 *(never used)* to 3 *(often used).*

For peer review only - http://bmjopen.bmj.com/site/about/guidelines.xhtml

The questionnaire also included the working environment scale-10 (WES-10) scale and a measure of workplace stressors [13]. The WES-10 is a 10-item scale that describes four aspects of the working environment; opportunity for personal and professional growth (self realisation); workload; interpersonal conflict; and nervousness [13]. Job stressors were assessed using a 15-item ED stressor scale (EDSS) [14]. Respondents were asked to rate on a scale of 1-15, how stressful they would find each of 15 stress-provoking events. They were also asked how often they experienced each event, ranging from 0 (*never*) to 3 (*daily*).

Patient and Public Involvement

This study did not include patients. As such, patients were not involved in the design or conduct of the study. Results will not be disseminated to patients directly.

Data analysis

Statistical analyses were performed in R version 3.5.1 [15]. Characteristics of responders were reported by site. A shortened JCS was then developed in three steps; 1) item reduction through review and consensus by content matter experts, 2) exploratory factor analysis (EFA) for further item reduction and to identify the factor structure of the revised scales, and 3) confirmatory factor analyses (CFA) to confirm the factors identified within the EFA.

EFA and CFA should not be conducted on the same sample as this results in over-fitting and optimistic estimates of model fit within the CFA [16]. As such, the sample was randomly split into equal sized derivation and validation cohorts. EFA was conducted on the derivation cohort to undertake further item reduction and to identify the factor structure of the reduced scale. Principal axis factoring with oblimin rotation was estimated using the R psych package [17]. As the data were ordinal, factor analysis was based on the polychoric correlation matrix [18]. The number of factors to be retained was identified based on a number of strategies. These included 1) the number of factors with eigenvalues greater than one, 2) examination of the scree plot to identify the number of factors where eigenvalues start to level off [19], 3) parallel analysis, where the number of factors to be retained reflects the factors where eigenvalues are higher than those computed from a simulated random dataset [20], and 4) interpretability of the factor solution. The Kaiser-Meyer-Olkin (KMO) Test for sampling adequacy was conducted to determine the suitability of items for factor analysis. Items with a value ≥0.70 show moderate suitability, while values ≥0.80 show good suitability for factor analysis [21]. Values <0.70 were considered for exclusion in the current study. Further item reduction was based on factor

For peer review only - http://bmjopen.bmj.com/site/about/guidelines.xhtml

loadings. Items that loaded highly (≥0.40) onto one component with low cross-loadings (≤0.20) on other factors were retained [19]. All other items were excluded, unless they were deemed to be an important coping strategy that was not captured by any retained items.

In the derivation cohort, there were 41 (9%) individuals with missing data on any of the JCS items, with the range of missing data for individual items being 1 (0.2% of respondents) to 11 missing responses (3% of respondents). There were no differences in median (or mean) JCS scores for those with and without missing data. As there were minimal missing data on any individual item, and few suitable methods for imputation of missing data in EFA [22], pairwise deletion of missing data was utilized for this analysis.

Once the final items and factor structure were identified through EFA, the validation cohort was utilized for CFA. CFA is a method of construct validation that is used when there is theory or research that posits an underlying latent variable structure [23]. All items comprising a particular subscale are loaded onto their related factor, and the acceptability of this model is evaluated by goodness of fit and strength of resulting parameter estimates [23]. A variety of goodness of fit measures were utilized in the current study to assess different aspects of model fit. These include absolute fit measures ($\chi^{2,}$ and the standardized root mean square residual [SRMR]), a parsimony correction index (the root mean square error of approximation [RMSEA], and comparative fit indices (the comparative fit index [CFI] and the Tucker-Lewis index [TLI]) [23]. Support for the target model is obtained where SRMR is close to 0.08 or below, RMSEA values are close to 0.06 or below, and CFI and TLI values are close to 0.90 or greater [24]. CFA was conducted in the lavaan R package [25].

Within the validation cohort, there were 37 respondents (8%) who had missing data on one or more JCS items. Missing data were imputed using ten datasets that were developed using the remaining JCS items, age, gender and country as predictors. Polychoric correlations were again utilised and the diagonally weighted least squares estimator was used to estimate model parameters [26]. Robust corrections were used to adjust for any non-independence of errors associated with clustering across sites. However, ICCs were very low for all items (Median = 2%, IQR=1 to 5%), suggesting that there was minimal site level variance in JCS items.

After the modified scale was developed, descriptive statistics for each of the scales were reported by country. Moreover, the criterion validity of the scale was established. Criterion

For peer review only - http://bmjopen.bmj.com/site/about/guidelines.xhtml

validity describes how well scores on one measure predict scores on another related measure [27]. Based on previous research, it was anticipated that maladaptive coping strategies would be associated with poorer perceptions of the work environment and high stress, while problem-focussed strategies would be associated with higher perceptions of the work environment and low stress. As such, the relationship between JCS subscales, the WES-10 subscales, and the EDSS were examined. For the EDSS, we focussed on the 5 stressors that are most commonly reported in the ED; Heavy workload, environmental concerns (e.g., overcrowding), inability to provide optimal care, high acuity patients, and workplace violence [28, 29]. Scatter plots showed that all associations were linear with no influential outliers. However, ICCs for several of the items were >5%, indicating site-level variance in these items. As such, generalized estimating equations were calculated regressing coping strategies on the WES subscales and EDSS. Each of these equations were calculated using a Gaussian distribution with an exchangeable working correlation.

Ethics Statement

This study was carried out in accordance with the code of ethics of the world medical association (Declaration of Helsinki). Hospital and University human research and ethics committees (HREC/14/QGC/173; NRS/15/16/HREC) approved the protocol for the Australian sites and the Regional Ethical Review Board in Linköping (permit number 2018/563-32) approved the protocol for the Swedish sites. Informed consent was implied through the completion and return of the survey.

RESULTS

Seventy-nine percent of the cohort was female, and the median age was 35 years. Respondents had worked in their current ED for a median of 5 years and half worked full time. Average time worked was 0.9 full time equivalent (FTE) (*SD*=0.2 FTE). Characteristics of the cohort by study site are provided in Table 1.

For peer review only - http://bmjopen.bmj.com/site/about/guidelines.xhtml

Table 1. Characteristics of the cohort

	Entire Cohort	ED 1	ED 2	ED 3	ED 4	ED 5	ED 6	ED 7	ED 8	ED 9	ED 10
Country	-	Sweden	Sweden	Sweden	Sweden	AUS	AUS	AUS	AUS	AUS	AUS
ED type		Large Urban	Tertiary	Small Urban	Large Urban	Small Urban	Large Urban	Large Urban	Tertiary	Tertiary	Large Urban
Number of surveys distributed	1709	126	145	44	108	196†		400	150	540†	
Total Responses	875	77	92	30	53	44	85	160	93	147	94
Response rate	51%	61%	63%	68%	49%	66%		40%	62%	45%	
Age, Median (IQR)	35 (28-45)	44 (32-50)	36 (28-45)	42 (33-52)	36.5 (29-50)	38 (33-45)	36 (30-45)	33 (27-45)	32 (26-40)	31 (26-41)	34 (27-43)
Female, n (%)	653 (79%)	66 (88%)	64 (74%)	29 (97%)	42 (81%)	28 (70%)	56 (74%)	119 (82%)	66 (73%)	108 (77%)	75 (83%)
Nurses, n (%)	641 (76%)	70 (97%)	63 (70%)	27 (90%)	40 (78%)	27 (63%)	53 (66%)	114 (75%)	58 (63%)	105 (73%)	84 (91%)
Years working in current ED, Median (IQR)	5 (2-10)	10 (6-20)	4 (2-8)	5 (4-8)	5 (1-13)	5 (2-11)	5 (2-10)	3 (1-7)	5 (2-10)	5 (2-10)	5 (2-10)
Years working as a clinician, Median (IQR)	9 (4-16)	18 (9-32)	10 (5-17)	17 (9-30)	10 (4-34)	11 (6-13)	11 (6-13)	7 (4-12)	8 (3-13)	6 (4-14)	8 (4-16)
Number of respondents working full time, n (%)	371 (47%)	53 (74%)	79 (88%)	20 (71%)	46 (90%)	16 (40%)	29 (36%)	50 (36%)	24 (28%)	37 (31%)	17 (19%)

AUS=Australia. †Some staff are employed across both sites

For peer review only

Content Matter Experts

The content matter experts were two of the study authors (JC and MW) who independently reviewed each item in terms of applicability to ED staff. There was initial agreement on 51 (85%) items. Where there was disagreement, this was discussed until consensus was achieved. This process identified 28 items that were not relevant to the ED context, resulting in a 32-item measure to be subject to factor analyses.

Exploratory factor analysis

The derivation sample comprised 437 respondents, with a median age of 33 (IQR 27-44), and 78% female. The KMO measure of the 32 JCS items revealed that 30 items were adequate for factor analysis, with the two items focusing on exercising and acceptance having low factorability. As there were remaining items within the JCS that assessed similar coping strategies to these 2 items, these were removed, leaving 30 items for exploratory factor analysis. Parallel analyses, and scree plots on the 30 items suggested that there were 3 to 6 factors underlying the items. Factor analyses with oblimin rotation were run using 3, 4, 5 and 6 factors. In each of these analyses, eight items had low loadings on all factors, or had low loadings with high cross loadings. These items focussed on worry, considering alternative methods for handing the situation, reframing the problem, getting mad, internalizing the problem, relaxation, distraction, and action planning. Again, these coping strategies were reflected in remaining JCS items and they were removed. The remaining 22-items fell on between 3 and 5-factors. However, the 4 and 5 factor solutions displayed factor splitting, with only a small number of items falling with low cross-loadings on the final factor. The 3-factor solution yielded interpretable factors. Based on the three-factor model, four additional items were removed (discussing the problem with significant others, objective perspective, blaming others, and minimizing the problem) for low loadings with high cross-loadings. All remaining items were retained as they were felt to be important for the scale. The final solution is provided in Table 2. The three factors were labeled problem-focussed coping (4 items), negative emotion-focussed coping (10-items), and positive emotion-focussed coping (4 items).

Table 2. Standardized factor loadings for factor structure outlined in exploratory factor analysis

Factors and Items†	Factor Loadings		
	NE	PE	PF

For peer review only - http://bmjopen.bmj.com/site/about/guidelines.xhtml

Negative Emotion-Focussed Coping			
3. Used smoking or medication for stress relief	**0.61**	0.01	0.06
9. Pessimistic thinking	**0.52**	-0.03	0.20
22. Spent time alone	**0.58**	0.15	-0.09
34. Drank	**0.46**	-0.03	0.09
46. Risky behaviour	**0.61**	0.04	0.05
48. Ignored problem	**0.58**	0.11	-0.25
51. Self-blame for problem	**0.51**	-0.12	0.16
53. Took stress-reducing medications	**0.61**	-0.10	0.20
56. Physical distancing	**0.80**	-0.06	-0.13
58. Wishful thinking	**0.49**	0.01	0.04
Positive Emotion-focussed Coping			
37. Hardiness attitude	0.14	**0.48**	0.08
39. Used humour	0.11	**0.49**	0.04
50. Optimistic thinking	-0.05	**0.82**	0.05
54. Refocus on good side	-0.02	**0.76**	-0.03
Problem-focussed coping			
15. Discussed problem with professional	-0.04	-0.07	**0.66**
27. Information seeking	0.07	0.13	**0.63**
42. Discussed problem with someone who has experienced the situation	0.00	0.13	**0.51**
45. Learned new skills	-0.04	0.26	**0.43**

†Items have been summarized. Complete items are from the JCS-A [10] © Anne Jalowiec
Bolded items are primary factor loading. NE=Negative emotion-focussed coping; PE=Positive emotion-focussed coping; PF=Problem-focussed coping

Confirmatory Factor Analysis

This factor structure identified through EFA was validated using confirmatory factor analysis on the remaining cohort. The validation sample comprised 438 individuals with a median age of 36 (IQR=28-46). Fitting the three-factor solution to the validation sample provided a good fit to the data (χ^2= 324, p <0.01, CFI=0.93, TLI=0.92, SRMR=0.08, RMSEA=0.06) and all items loaded significantly onto their respective factors. Standardized loadings are provided in Table 3.

For peer review only - http://bmjopen.bmj.com/site/about/guidelines.xhtml

Table 3. Standardized factor loadings for theorised 3-factor model in confirmatory factor analysis

Factor and Items†	Factor Loadings		
	NE	PE	PF
**Negative Emotion-Focussed Coping (NE)**			
3. Used smoking or medications for stress relief	0.44		
9. Pessimistic thinking	0.38		
22. Spent time alone	0.63		
34. Drank	0.46		
46. Risky behaviour	0.57		
48. Ignored problem	0.63		
51. Self-blame for problem	0.63		
53. Took stress-reducing medications	0.52		
56. Physical distancing	0.76		
58. Wishful thinking	0.60		
**Positive Emotion-focussed Coping (PE)**			
37. Hardiness attitude		0.55	
39. Used humour		0.57	
50. Optimistic thinking		0.80	
54. Refocus on good side		0.83	
**Problem-focussed coping (PF)**			
15. Discussed problem with professional			0.45
27. Information seeking			0.45
42. Discussed problem with someone who has experienced the situation			0.58
45. Learned new skills			0.56

†Items have been summarized. Complete items are from the JCS-A [10] © Anne Jalowiec
NE=Negative emotion-focussed coping; PE=Positive emotion-focussed coping; PF=Problem-focussed coping

Descriptive Data and Criterion Validity

Descriptive data for the overall cohort and by country is reported in Table 4. Negative emotion-focussed coping strategies were used less often than positive emotion-focussed coping or

For peer review only - http://bmjopen.bmj.com/site/about/guidelines.xhtml

problem-focussed coping strategies. The cohort from Sweden reported slightly less use of negative coping strategies and slightly more use of positive and problem-focussed strategies.

Table 4: Coping strategies by country

	Overall Cohort	Australia	Sweden	Difference (95% CI of difference)
Negative Emotion-Focussed Coping	1.0 (0.5)	1.1 (0.5)	0.8 (0.4)	-0.3 (-0.3 to 0.2)
Positive Emotion-Focussed Coping	2.1 (0.6)	2.1 (0.5)	2.3 (0.6)	0.2 (0.1 to 0.3)
Problem-focussed Coping	1.9 (0.6)	1.9 (0.6)	2.1 (0.5)	0.2 (0.1 to 0.3)

The scale ranges from 0 (never used) to 3 (often used). CI=confidence interval

Coefficients from the regression of coping strategies on WES-10 subscales are provided in Table 5. There was an association between the use of negative emotion-focussed coping strategies and poor perceptions of the work environment (lower opportunity for personal and professional growth, higher workload, higher conflict among staff members and higher nervousness or tension about going to work). The use of either problem-focussed coping strategies or positive emotion-focussed coping strategies had a weak association with perceptions that the workplace provided opportunity for personal and professional growth (self-realisation).

Coefficients from the regression of coping strategies on ratings of stressors are provided in Table 5. Respondents reporting higher use of negative emotion-focussed coping strategies rated events to be more stressful. There was no association between ratings of stressors and either positive emotion- focussed coping or problem-focussed coping. One exception was that higher problem-focussed coping had a weak relationship with higher stress over inability to provide optimum care.

Table 5. Regression coefficients and 95% confidence intervals from coping strategies with WES-10 subscales and job stressors.

For peer review only - http://bmjopen.bmj.com/site/about/guidelines.xhtml

	Negative Emotion-Focussed Coping	Positive Emotion-Focussed Coping	Problem-Focussed Coping
WES-10 subscales			
Self Realisation	-0.3 (-0.4 to -0.2)	0.2 (0.1 to 0.2)	0.1 (0.1 to 0.2)
Workload	0.3 (0.2 to 0.3)	0 (0 to 0.1)	0.1 (0 to 0.2)
Conflict	0.5 (0.4 to 0.6)	0 (-0.1 to 0.1)	0.1 (0 to 0.2)
Nervousness	0.6 (0.5 to 0.7)	-0.1 (-0.2 to 0)	0 (-0.1 to 0.1)
ED SS			
Workplace violence	0.9 (0.4 to 1.4)	0.2 (-0.3 to 0.6)	0.2 (-0.2 to 0.7)
Heavy workload	1.5 (1.1 to 1.8)	-0.2 (-0.6 to 0.1)	0.2 (-0.2 to 0.5)
High acuity patients	1.4 (1.0 to 1.8)	-0.3 (-0.7 to 0.1)	0 (-0.4 to 0.4)
Inability to provide optimum care	1.5 (1.1 to 1.9)	0 (-0.3 to 0.4)	0.7 (0.3 to 1.1)
Environmental concerns	1.6 (1.1 to 2.0)	-0.1 (-0.5 to 0.3)	0.4 (0.0 to 0.8)

WES=work environment scale (10 items). ED SS = Emergency Department stressor scale. The coefficient represents the predicted change in WES-10 scores or ED stressor scales for each 1-point increase in coping strategies.

DISCUSSION

This study utilised data from a large sample of international ED staff to develop a refined JCS for future use by ED staff. The final scale incorporated 18 items measuring three styles of coping; problem-focussed coping, positive emotion-focussed coping, and negative emotion-focussed coping. It is short enough to encourage inclusion in future surveys exploring ED staff perceptions. Data exploration using this tool indicated that the use of negative coping strategies was associated with poor perceptions of the work environment and higher ratings of ED job stressors in both Australian and Swedish EDs. This modified scale is likely to be useful in identifying the types of coping strategies employed by ED staff and for use in developing and evaluating interventions to reduce the use of maladaptive coping strategies.

The three categories of coping identified in the present study; problem-focussed coping, positive emotion-focussed coping, and negative emotion-focussed coping aligned with other theoretical literature. For example, the division of problem and emotion-focussed coping accords with

For peer review only - http://bmjopen.bmj.com/site/about/guidelines.xhtml

Lazarus's theoretical model of coping [5]. This categorization also acknowledges that there are different styles of emotion-focussed coping, with some linked with higher distress [7]. By developing a shortened JCS that corresponds with theoretical and empirical research on coping, we have not only developed an ED based tool to explore coping strategies, but also a tool that is likely to be suitable for administration more broadly in the busy environments experienced by acute care health professionals. However, further validation would be required to identify broader utility of this scale.

Using the modified JCS enabled the team to establish an association between negative emotion-focused coping and poor perceptions of the working environment, as well as higher ratings of stress around listed events. This pattern of response is consistent with previous research [3, 8, 30] and accords with pragmatic perceptions of the development of burnout in ED staff. Maladaptive coping strategies have been consistently linked to poor perceptions of stress management [30], poor psychological health [3] and burnout [8]. We also found that problem-focused and positive emotion-focused strategies were largely unrelated to perceptions of stress around specific events. This differs from a number of previous studies finding that problem-focused strategies in particular have beneficial outcomes, such as lower burnout and distress [3, 8]. However, the broader literature does note that problem- and emotion-focused strategies may not be classed as inherently good or bad [5]. Instead, their effectiveness depends on the situation [7]. In situations where stressors are controllable (e.g., taking an exam), problem-focused coping is useful [31]. Conversely, when dealing with stressors such as a major loss, it may be adaptive initially to engage in some palliative coping [32]. As such, these strategies may not show clear relationships with broad outcome variables.

The strengths of this study include the generation of a validated revised JCS using a large multidisciplinary international sample. The limitations include the lack of availability of longitudinal data, meaning that test-retest reliability was unable to be determined. Test-retest reliability is necessary to ensure that the scale is reliable and stable in assessing coping strategies across time. The response rate was reasonable for a survey design. However, it is unknown whether the respondents were representative of the broader population. No data were available on burnout or employee mental health and so limited assessment of the outcomes of coping could be conducted. The JCS assesses coping using a 4-item Likert scale. Research has shown that the reliability, validity and discriminating power of four-point scale is relatively poor, with the optimum

For peer review only - http://bmjopen.bmj.com/site/about/guidelines.xhtml

number of categories being between 7 to 10 [33]. Future research may benefit from incorporating a larger number of response categories.

The ED can be a particularly stressful workplace. Stressors can result in increased sick leave and staff burnout, poor staff recruitment and retention, decreased staff morale, and decreased job satisfaction. Understanding ways in which ED staff cope with stressors that pertain to their work is important. We have developed a revised JCS to assess the coping strategies utilised by ED staff. This shortened version of the JCS can identify maladaptive and positive coping strategies and may be used to evaluate the outcome of interventions that seek to promote positive coping strategies.

For peer review only

FUNDING

This study was supported by an Emergency Medicine Foundation grant (EMSS-410R22-2014)

CONFLICTS OF INTEREST

The authors declare no competing interests

AUTHOR STATEMENT

JG, MW, AJ, EC, DW, OT, LA and JC conceived and designed the study. MW, AJ, EC, DW, OT, LA and JC. were involved in acquisition of data. JG analysed the data. MW, AJ, EC, DW, and JC assisted with interpretation of the data. JG, MW and JC drafted the work and all authors revised the manuscript critically for important intellectual content.

DATA STATEMENT

There are no additional data available

REFERENCES

1 Cronin G, Cronin C. Why does A&E attract newly qualified registered nurses? Accident and Emergency Nursing 2006;14:71-7.
2 Rozo JA, Olson DM, Thu H, et al. Situational factors associated with burnout among emergency department nurses. Workplace Health Saf 2017;65(6):262-5.
3 Yates PJ, Benson EV, Harris A, et al. An investigation of factors supporting the psychological health of staff in a UK emergency department. Emerg Med J 2012;29:533-5.

For peer review only - http://bmjopen.bmj.com/site/about/guidelines.xhtml

4 Sawatzky JV, Enns CL. Exploring the key predictors of retention in emergency nurses. J Nurs Manag 2012;20:696-707.
5 Lazarus RS, Folkman S. Stress, appraisal, and coping. New York: Springer; 1984.
6 Folkman S, Lazarus RS. Coping as a mediator of emotion. J Pers Soc Psycho 1988;54(3):466-75.
7 Folkman S, Moskowitz JT. Coping: Pitfalls and Promise. Ann Rev Psychol 2004;2004(55):745-74.
8 Howlett M, Doody K, Murray J, et al. Burnout in emergency department healthcare professionals is associated with coping style: a cross-sectional survey. Emerg Med J 2015;32:722-7.
9 Donoghue KJ. Measuring coping: Evaluating the psychometric properties of the COPE: Edith Cowan Univeristy; 2004.
10 Jalowiec A. The Jalowiec Coping Scale. In: Strickland O, editor. Measurement of Nursing Outcomes New York: Springer; 2003. p. 71–97.
11 Jalowiec A. Jalowiec Coping Scale: Overview of JCS by Dr. Anne Jalowiec 2001. http://www.allcare.org/CancerPain-and-SymptomManagement/wellbe/wbm4/Jalowiec%20coping%20scale%20overview.pdf. (accessed
12 Ulvik B, Johnsen TB, Nygard O, et al. Factor structure of the revised Jalowiec Coping Scale in patients admitted for elective coronary angiography. Scand J Caring Sci 2008;22(4):596-607.
13 Røssberg JI, Eiring Ø, Friis S. Work environment and job satisfaction: A psychometric evaluation of the Working Environment Scale-10. Soc Psychiatry Psychiatr Epidemiol 2004;39:576-80.
14 Ross-Adjie GM, Leslie G, Gillman L. Occupational stress in the ED: What matters to nurses? Australas Emerg Nurs J 2007;10:117-23.
15 R Core Team. R: A language and environment for statistical computing. Vienna, Austria: R Foundation for Statistical Computing; 2018.
16 Fokkema M, Greiff S. How performing PCA and CFA on the same data equals trouble: overfitting in the assessment of internal structre and some editorial thoughts on it. Eur J Psychol Assess 2017;33(6):399-402.
17 Revelle W. psych: Procedures for Psychological, Psychometric, and Personality Research. Evanston, Illinois: Northwestern Univeristy; 2018.
18 Lee C-T, Zhang G, Edwards MC. Exploratory least squares estimation of parameters in exploratory factor analysis with ordinal data. Multivariate Behav Res 2012;47(2):314-39.
19 Matsunaga M. How to Factor-Analyze Your Data Right: Do's, Don'ts, and How-To's. Int J Psychol Res 2010;3(1):97-110.
20 Horn JL. A rationale and test for the number of factors in factor analysis. Psychometrika 1965;30(2):1790185.
21 Kaiser H. An index of factor simplicity. Psychometrika 1974;39:31-6.
22 Çokluk O, Kayri M. The Effects of Methods of Imputation for Missing Values on the Validity and Reliability of Scales. Educational Sciences: Theory and Practice 2011;11(1):303-9.
23 Brown TA. Confirmatory factor analysis for applied research, second edition. New York: Guilford Publications; 2015.
24 Hu L-t, Bentler PM. Cutoff criteria for fit indexes in covariance structure analysis: Conventional criteria versus new alternatives. Struct Equ Modeling 1999;6:1-55.
25 Rosseel Y. lavaan: An R package for structural equation modeling. J Stat Softw 2012;48(2):1-36.
26 Li C-H. The performance of ML, DWLS, and ULS estimation with robust corrections in structural equation models with ordinal variables. Psychol Methods 2016;21(3):369-87.
27 Borneman MJ. Criterion Validity. In: Salkind NJ, editor. Encyclopedia of Research Design: SAGE; 2010.
28 Elder EG, Johnston A, Wallis M, et al. Emergency clinician perceptions of occupational stressors and coping strategies: a multi-site study. Int Emerg Nurs 2018.
29 Xu H, Johnston A, Greenslade JH, et al. Stressors and coping strategies of emergency department nurses and doctors: A cross-sectional study. Australasian Emergency Care 2018.

For peer review only - http://bmjopen.bmj.com/site/about/guidelines.xhtml

30 Holton MK, Barry AE, Chaney D. Employee stress management: An examination of adaptive and maladaptive coping strategies on employee health. Work 2016;53:299-305.
31 Folkman S, Lazarus RS. If it changes it must be a process: study of emotion and coping during three stages of a college examination. J Pers Soc Psycho 1985;48:150-70.
32 Stroebe MS, Schut H, editors. Meaning making in the dual process model of coping with bereavement. Washington, DC: American Psychological Association; 2001.
33 Preston CC, Colman AM. Optimal number of response categories in rating scales: reliability, validity, discriminating power, and respondent preferences. Acta Psychol 2000;104:1-15.

.

For peer review only

For peer review only - http://bmjopen.bmj.com/site/about/guidelines.xhtml

STROBE 2007 (v4) Statement—Checklist of items that should be included in reports of *cross-sectional studies*

Section/Topic	Item #	Recommendation	Reported on page #
**Title and abstract**	1	(*a*) Indicate the study's design with a commonly used term in the title or the abstract	1
		(*b*) Provide in the abstract an informative and balanced summary of what was done and what was found	3
**Introduction**			
Background/rationale	2	Explain the scientific background and rationale for the investigation being reported	5
Objectives	3	State specific objectives, including any prespecified hypotheses	6
**Methods**			
Study design	4	Present key elements of study design early in the paper	6
Setting	5	Describe the setting, locations, and relevant dates, including periods of recruitment, exposure, follow-up, and data collection	6
Participants	6	(*a*) Give the eligibility criteria, and the sources and methods of selection of participants	6
Variables	7	Clearly define all outcomes, exposures, predictors, potential confounders, and effect modifiers. Give diagnostic criteria, if applicable	6-7
Data sources/ measurement	8*	For each variable of interest, give sources of data and details of methods of assessment (measurement). Describe comparability of assessment methods if there is more than one group	6-7
Bias	9	Describe any efforts to address potential sources of bias	N/A
Study size	10	Explain how the study size was arrived at	6
Quantitative variables	11	Explain how quantitative variables were handled in the analyses. If applicable, describe which groupings were chosen and why	7-9
Statistical methods	12	(*a*) Describe all statistical methods, including those used to control for confounding	7-9
		(*b*) Describe any methods used to examine subgroups and interactions	N/A
		(*c*) Explain how missing data were addressed	8
		(*d*) If applicable, describe analytical methods taking account of sampling strategy	N/A
		(*e*) Describe any sensitivity analyses	N/A
**Results**			

For peer review only

Participants	13*	(a) Report numbers of individuals at each stage of study—eg numbers potentially eligible, examined for eligibility, confirmed eligible, included in the study, completing follow-up, and analysed	6
		(b) Give reasons for non-participation at each stage	N/A
		(c) Consider use of a flow diagram	N/A
Descriptive data	14*	(a) Give characteristics of study participants (eg demographic, clinical, social) and information on exposures and potential confounders	10
		(b) Indicate number of participants with missing data for each variable of interest	8
Outcome data	15*	Report numbers of outcome events or summary measures	N/A
Main results	16	(*a*) Give unadjusted estimates and, if applicable, confounder-adjusted estimates and their precision (eg, 95% confidence interval). Make clear which confounders were adjusted for and why they were included	11-14
		(*b*) Report category boundaries when continuous variables were categorized	N/A
		(*c*) If relevant, consider translating estimates of relative risk into absolute risk for a meaningful time period	N/A
Other analyses	17	Report other analyses done—eg analyses of subgroups and interactions, and sensitivity analyses	N/A
**Discussion**			
Key results	18	Summarise key results with reference to study objectives	15-16
Limitations	19	Discuss limitations of the study, taking into account sources of potential bias or imprecision. Discuss both direction and magnitude of any potential bias	16
Interpretation	20	Give a cautious overall interpretation of results considering objectives, limitations, multiplicity of analyses, results from similar studies, and other relevant evidence	17
Generalisability	21	Discuss the generalisability (external validity) of the study results	16
**Other information**			
Funding	22	Give the source of funding and the role of the funders for the present study and, if applicable, for the original study on which the present article is based	18

*Give information separately for cases and controls in case-control studies and, if applicable, for exposed and unexposed groups in cohort and cross-sectional studies.

Note: An Explanation and Elaboration article discusses each checklist item and gives methodological background and published examples of transparent reporting. The STROBE checklist is best used in conjunction with this article (freely available on the Web sites of PLoS Medicine at http://www.plosmedicine.org/, Annals of Internal Medicine at http://www.annals.org/, and Epidemiology at http://www.epidem.com/). Information on the STROBE Initiative is available at www.strobe-statement.org.

For peer review only

BMJ Open

Development of a revised Jalowiec coping scale for use by emergency clinicians: A cross-sectional scale development study

Journal:	*BMJ Open*
Manuscript ID	bmjopen-2019-033053.R1
Article Type:	Original research
Date Submitted by the Author:	09-Oct-2019

Complete List of Authors:	Greenslade, Jaimi; Queensland University of Technology Faculty of Health, Institute of Health and Biomedical Innovation, School of Public Health annd Social Work; Royal Brisbane and Women's Hospital, Emergency and Trauma Centre<br>Wallis, Marianne; University of the Sunshine Coast, School of Nursing & Midwifery<br>Johnston, Amy; Princess Alexandra Hospital Health Service District, Department of Emergency Medicine; University of Queensland - Saint Lucia Campus, School of Nursing, Midwifery, and Social Work<br>Carlström, Eric; Goteborgs Universitet,<br>Wilhelms, Daniel; Linköping University, Department of Health and Medical Sciences,; Local Health Care Services in Central Östergötland, Department of Emergency Medicine<br>Thom, Ogilvie; Sunshine Coast Hospital and Health Service, Department of Emergency Medicine<br>Abraham, Louisa; Royal Brisbane and Women's Hospital,<br>Crilly, Julia; Griffith University, Menzies Health Institute; Goldcoast Health Service District, Department of Emergency Medicine

**Primary Subject Heading**:	Emergency medicine
Secondary Subject Heading:	Mental health
Keywords:	ACCIDENT & EMERGENCY MEDICINE, Human resource management < HEALTH SERVICES ADMINISTRATION & MANAGEMENT, MENTAL HEALTH

SCHOLARONE™ Manuscripts

DEVELOPMENT OF A REVISED JALOWIEC COPING SCALE FOR USE BY EMERGENCY CLINICIANS: A CROSS-SECTIONAL SCALE DEVELOPMENT STUDY

AUTHORS

Associate Professor Jaimi H GREENSLADE, B.Psych (Hons), M.Sci (Stats), PhD

Emergency and Trauma Centre, Royal Brisbane and Women's Hospital, Herston, QLD, 4029, Australia

Australian Centre for Health Services Innovation, School of Public Health and Social Work, Institute of Health and Biomedical Innovation, Queensland University of Technology, Kelvin Grove, QLD, 4059, Australia

Professor Marianne WALLIS, RN, BSc (Hons), PhD

Sunshine Coast Health Institute, University of the Sunshine Coast, Sippy Downs, QLD, 4558, Australia

Menzies Health Institute Queensland, Griffith University, QLD, 4222, Australia

Dr Amy N.B. JOHNSTON BN, BSc (Hons), MEd, PhD

Department of Emergency Medicine, Princess Alexandra Hospital, QLD, 4102, Australia

School of Nursing, Midwifery, and Social Work, The University of Queensland, QLD, 4072, Australia

Professor Eric CARLSTRÖM, RN, PhD

Health and Crisis Management and Policy, Sahlgrenska Academy, University of Gothenburg Gothenburg, Sweden

University College of Southeast Norway, Vestfold, Norway

Associate Professor Daniel B WILHELMS, MD, PhD

Department of Health and Medical Sciences, Linköping University, Linköping, Sweden

Department of Emergency Medicine, Local Health Care Services in Central Östergötland, Region Östergötland,Linköping, Sweden

Dr Ogilvie THOM, MBBS, FACEM

For peer review only - http://bmjopen.bmj.com/site/about/guidelines.xhtml

Department of Emergency Medicine, Sunshine Coast Hospital and Health Service, Queensland, 4560, Australia.

Dr Louisa ABRAHAM, MBChB, BSc (Hons)
Emergency and Trauma Centre, Royal Brisbane and Women's Hospital, Herston, QLD, 4029, Australia

Professor Julia CRILLY BN, MN (Hons), PhD
Department of Emergency Medicine, Gold Coast Health, QLD, 4215, Australia
Menzies Health Institute Queensland, Griffith University, QLD, 4222, Australia

On behalf of the WES investigators
Hui (Grace) XU, MNP
Department of Emergency Medicine, Logan Hospital

Elizabeth ELDER, MN
School of Nursing and Midwifery, Griffith University

James HUGHES, PhD
Emergency and Trauma Centre, Royal Brisbane and Women's Hospital
School of Nursing, Queensland University of Technology

Monica A MAGNUSSON, MScN
Emergency Department, Linköping University Hospital, Linköping

CORRESPONDING AUTHOR
Associate Professor Jaimi H GREENSLADE
Institute of Health and Biomedical Innovation, School of Public Health and Social Work
Queensland University of Technology
Musk Avenue
KELVIN GROVE, QLD, 4059, Australia
j.greenslade@qut.edu.au
Ph: +61 7 3646 6262

For peer review only

For peer review only - http://bmjopen.bmj.com/site/about/guidelines.xhtml

WORD COUNT: 3958

KEY WORDS: Coping; Emergency Departments; Scale Development; Stress

ABSTRACT

Objectives: The aim of this study was to develop and validate a scale to measure the coping strategies used by emergency staff in response to workplace stress. To achieve this aim, we developed a refined Jalowiec Coping Scale, termed the Jalowiec coping scale – Emergency Department (JCS-ED) and validated this scale on a sample of emergency clinicians.

Design: A cross-sectional survey incorporating the Jalowiec coping scale, the working environment scale-10 (WES-10) and a measure of workplace stressors was administered between July 2016 and June 2017. The JCS-ED was developed in three stages 1) item reduction through content matter experts, 2) exploratory factor analysis for further item reduction and to identify the factor structure of the revised scale, and 3) confirmatory factor analyses to confirm the factors identified within the exploratory factor analysis.

Setting: Six Emergency Departments (EDs) in Australia and four in Sweden. There were three tertiary hospitals, five large-urban hospitals and two small urban hospitals.

Participants: Participants were eligible for inclusion if they worked full-time or part-time as medical or nursing staff in the study EDs. The median age of participants was 35 years (IQR: 28-45 years) and they had been working in the ED for a median of 5 years (IQR: 2-10 years). Seventy-nine percent were female and 76% were nurses.

Results: A total of 875 ED staff completed the survey (response rate 51%). The content matter experts reduced the 60-item scale to 32 items. Exploratory factor analyses then further reduced the scale to 18 items assessing three categories of coping; problem-focussed coping, positive emotion-focussed coping, and negative emotion-focussed coping. Confirmatory factor analysis supported this 3-factor structure. Negative coping strategies were associated with poor perceptions of the work environment and higher ratings of stress.

For peer review only - http://bmjopen.bmj.com/site/about/guidelines.xhtml

Conclusions: The JCS-ED assesses maladaptive coping strategies along with problem- and emotion-focussed coping styles. It is a short instrument that is likely to be useful in measuring the types of coping strategies employed by staff.

STRENGTHS AND LIMITATIONS OF THE STUDY

- This study utilizes a large cohort of nurses and physicians from 10 Emergency Departments in two countries.
- This coping scale has been validated for use in the emergency department and may also be relevant to the broader range of staff employed in an acute care setting.
- The JCS-ED is practical as it is short enough to encourage inclusion in future surveys exploring ED staff perceptions.
- No longitudinal data were available, meaning that test-retest reliability was unable to be determined
- The response rate was reasonable for a survey design. However, it is unknown whether there was non-response bias.

For peer review only

For peer review only - http://bmjopen.bmj.com/site/about/guidelines.xhtml

INTRODUCTION

The Emergency Department (ED) is a dynamic and demanding environment. Positive factors such as a supportive team environment, the development of high-level clinical skills, and the challenges around dealing with varied patient groups attract clinical staff to this setting [1]. However, high workload, inadequate staffing, workplace violence, and dealing with critically ill patients can place strain on employees [2]. Indeed, high levels of psychological distress [3] and staff turnover [4] are evident amongst ED staff. The coping strategies employed by clinicians may be one critical component in distinguishing these contrasting views of the ED as a stressful versus positive environment.

Coping is defined as the thoughts and behaviours used to manage the demands of situations that are appraised as stressful [5]. The transactional model of stress and coping outlines the processes by which stressful situations give rise to coping behaviours, and ultimately to workplace well-being outcomes [5]. Specifically, this model posits that individuals appraise or evaluate their situation. If a situation is deemed taxing or overwhelming, they will engage in thoughts or actions (coping strategies) to manage that situation [6, 7]. Many different coping strategies can be employed, and these depend on both the environment and on personal disposition [8]. Such coping strategies serve to alter stress in various ways. First, they may address the problem causing distress (problem-focussed coping) [8]. Problem-focussed coping attempts to remove the source of distress, thereby removing the stressor [6]. As such, individuals who successfully utilise problem-focussed coping experience lower distress and, thus, experience fewer negative outcomes from stress [6]. Second, coping strategies may attempt to ameliorate the negative emotions associated with the stressor (emotion-focussed coping) [8]. Emotion focussed coping can change the way we think about or interpret what is happening [6]. Some emotion focussed coping strategies may be positive and functional, while others can have negative consequences [8]. For example, drinking to cope may provide short term relief from stress, but does not actually reduce the problem in the longer term. Such maladaptive coping does not reduce the impact of the stressful situation and, thus, is linked to poorer outcomes [8]. In line with this theory, previous studies have suggested that ED staff using problem-focussed coping have lower levels of burnout [9] and better psychological health [3] than those using emotion-oriented coping or maladaptive coping strategies. Identifying the functional and maladaptive coping strategies that are used within the ED is important as these

For peer review only - http://bmjopen.bmj.com/site/about/guidelines.xhtml

are amenable to intervention, with therapies such as cognitive behaviour therapy being shown to successfully increase the use of beneficial strategies [8].

One major challenge in identifying and modifying coping strategies within the ED is that the objective measurement of coping has been difficult. There are no validated scales of which we are aware that were designed to measure coping within the ED. As such, various generic coping scales have been applied to this setting [e.g 9, 10, 11, 12]. Existing scales are lengthy [8], or have displayed poor psychometric properties [13]. For example, the COPE questionnaire is the most commonly used instrument in adult samples [14]. This questionnaire is a 53-item index, but has been found to have an equivocal factor structure with moderate reliability. [14, 15]. The Jalowiec Coping Scale (JCS) is another coping scale, with the advantage that it was specifically developed to measure the process of coping in a healthcare setting. This scale incorporates 60 items distributed across 8 dimensions and has been translated into more than 20 languages [16]. The JCS was originally developed to measure problem- and emotion-focused coping styles, but the final version identifies 8 different dimensions of coping. The JCS has been used nationally and internationally with both patients and employees in the healthcare setting [17]. While this scale has been extensively utilized, studies assessing its psychometric properties have not supported the 8-item structure [18]. Further, a 60-item scale is arguably too long for busy ED staff.

The overarching goal of this study was to produce a coping scale that would be useful for assessing coping in ED staff. This instrument should incorporate items to identify beneficial and maladaptive problem- and emotion-focused coping strategies while also being short enough for easy completion in a busy work environment. To achieve this goal, we sought to develop and validate a modified JCS coping scale for the emergency department setting.

METHODS

Participants and Design

All full-time and part-time medical and nursing staff employed in the study EDs were eligible for inclusion in this study. There were no exclusion criteria. A cross-sectional paper survey was administered between July 2016 and June 2017 depending on logistics at each site. Surveys were hand distributed to 1709 staff by a local investigator at each site, with 876 returned. One survey was excluded due to extensive missing data, making the final sample size 875 (51% response rate). Staff were also provided with information and invited to participate via email

For peer review only - http://bmjopen.bmj.com/site/about/guidelines.xhtml

and in ward-based information sessions. No compensation was provided to staff for completing the survey. Surveys were returned to locked boxes within each hospital ED, or via stamped self-addressed envelopes. A reminder email was sent out two-weeks after survey distribution.

Instrument

The JCS was used to assess the coping strategies employed by ED clinicians. The JCS incorporates 60-items that were developed to measure eight different coping dimensions. These include facing up to the problem (confrontive coping), avoiding the problem (evasive coping), positive thinking (optimistic coping), pessimistic thinking (fatalistic coping), releasing emotions (emotive coping), making yourself feel better (palliative coping), using support systems (supportive coping), and depending on yourself (self-reliant coping). Respondents report how often they have used each coping method, with scores for each item ranging from 0 *(never used)* to 3 *(often used).*

The questionnaire also included the working environment scale-10 (WES-10) scale and a measure of workplace stressors [19]. The WES-10 is a 10-item scale that describes four aspects of the working environment; opportunity for personal and professional growth (self-realization, 4 items); workload (2 items); interpersonal conflict (2 items); and nervousness (2 items) [19]. Respondents are asked to answer how they feel about each item on 1 to 5 scale, with Likert-scale labels differing according to the question asked. This scale has been used within an ED [20], but was not specifically developed or validated in this setting. Cronbach alpha for self-realization was 0.72 while Spearman-Brown was 0.64 for workload, 0.57 for conflict and 0.70 for nervousness. These show moderate internal consistency and are in line with reliability coefficients reported in previous studies [19, 20]. Job stressors were assessed using a 15-item ED stressor scale (EDSS) [21]. This scale was designed to assesses stressors reported by nurses within an Australian ED [21]. Respondents were asked to rate on a scale of 1-15, how stressful they would find each of 15 stress-provoking events. They were also asked how often they experienced each event, ranging from 0 (*never*) to 3 (*daily*). Items on this scale are not combined to form sub-scales, they each assess a different stressor within the ED.

Patient and Public Involvement

This study did not include patients. As such, patients were not involved in the design or conduct of the study. Results will not be disseminated to patients directly.

For peer review only - http://bmjopen.bmj.com/site/about/guidelines.xhtml

Data analysis

Statistical analyses were performed in R version 3.5.1 [22]. Characteristics of responders were reported by site. A shortened JCS was then developed in three steps. First, item reduction was conducted through review and consensus by content matter experts. Second exploratory factor analysis (EFA) was conducted to reduce the number of items and to identify the factor structure of the revised scales. For this step, principal axis factoring with oblimin rotation was estimated using the R psych package [23]. As the data were ordinal, factor analysis was based on the polychoric correlation matrix [24]. Third, once the final items and factor structure were identified through EFA, CFA was conducted. CFA is a method of construct validation that is used when there is theory or research that posits an underlying latent variable structure [25]. All items comprising a particular subscale are loaded onto their related factor, and the acceptability of this model is evaluated by goodness of fit measures and the strength of resulting parameter estimates [25]. CFA was conducted in the lavaan R package [26].

After the modified scale was developed, descriptive statistics for each of the scales were reported by country. Moreover, the criterion validity of the scale was established. Criterion validity describes how well scores on one measure predict scores on another related measure [27]. Based on previous research, it was anticipated that maladaptive coping strategies would be associated with poorer perceptions of the work environment and high stress, while problem-focussed strategies would be associated with higher perceptions of the work environment and low stress. As such, the relationship between JCS subscales, the WES-10 subscales, and the EDSS were examined. For the EDSS, we focussed on the 5 stressors that are most commonly reported in the ED; Heavy workload, environmental concerns (e.g., overcrowding), inability to provide optimal care, high acuity patients, and workplace violence [28, 29]. Scatter plots showed that all associations were linear with no influential outliers. However, ICCs for several of the items were >5%, indicating site-level variance in these items. As such, generalized estimating equations were calculated regressing coping strategies on the WES subscales and EDSS. Each of these equations were calculated using a Gaussian distribution with an exchangeable working correlation.

Ethics Statement

This study was carried out in accordance with the code of ethics of the world medical association (Declaration of Helsinki). Hospital and University human research and ethics committees (HREC/14/QGC/173; NRS/15/16/HREC) approved the protocol for the Australian

For peer review only - http://bmjopen.bmj.com/site/about/guidelines.xhtml

sites and the Regional Ethical Review Board in Linköping (permit number 2018/563-32) approved the protocol for the Swedish sites. Informed consent was implied through the completion and return of the survey.

RESULTS

Seventy-nine percent of the cohort was female, and the median age was 35 years. Respondents had worked in their current ED for a median of 5 years and half worked full time. Average time worked was 0.9 full time equivalent (FTE) (*SD*=0.2 FTE). Characteristics of the cohort by study site are provided in Table 1. For the nursing cohort, the average age and proportion of females is similar to data available from national surveys in both Australia [30] and Sweden [31]. Similarly, the age and sex of physicians were similar to data reported in Australia [32], but less information is available from Sweden.

For peer review only

For peer review only - http://bmjopen.bmj.com/site/about/guidelines.xhtml

Table 1. Characteristics of the cohort

	Entire Cohort	ED 1	ED 2	ED 3	ED 4	ED 5	ED 6	ED 7	ED 8	ED 9	ED 10
Country	-	Sweden	Sweden	Sweden	Sweden	AUS	AUS	AUS	AUS	AUS	AUS
ED type		Large Urban	Tertiary	Small Urban	Large Urban	Small Urban	Large Urban	Large Urban	Tertiary	Tertiary	Large Urban
Number of surveys distributed	1709	126	145	44	108	196†		400	150	540†	
Total Responses	875	77	92	30	53	44	85	160	93	147	94
Response rate	51%	61%	63%	68%	49%	66%		40%	62%	45%	
Age, Median (IQR)	35 (28-45)	44 (32-50)	36 (28-45)	42 (33-52)	36.5 (29-50)	38 (33-45)	36 (30-45)	33 (27-45)	32 (26-40)	31 (26-41)	34 (27-43)
Female, n (%)	653 (79%)	66 (88%)	64 (74%)	29 (97%)	42 (81%)	28 (70%)	56 (74%)	119 (82%)	66 (73%)	108 (77%)	75 (83%)
Nurses, n (%)	641 (76%)	70 (97%)	63 (70%)	27 (90%)	40 (78%)	27 (63%)	53 (66%)	114 (75%)	58 (63%)	105 (73%)	84 (91%)
Years working in current ED, Median (IQR)	5 (2-10)	10 (6-20)	4 (2-8)	5 (4-8)	5 (1-13)	5 (2-11)	5 (2-10)	3 (1-7)	5 (2-10)	5 (2-10)	5 (2-10)
Years working as a clinician, Median (IQR)	9 (4-16)	18 (9-32)	10 (5-17)	17 (9-30)	10 (4-34)	11 (6-13)	11 (6-13)	7 (4-12)	8 (3-13)	6 (4-14)	8 (4-16)
Number of respondents working full time, n (%)	371 (47%)	53 (74%)	79 (88%)	20 (71%)	46 (90%)	16 (40%)	29 (36%)	50 (36%)	24 (28%)	37 (31%)	17 (19%)

AUS=Australia. †Some staff are employed across both sites

For peer review only

Content Matter Experts

The content matter experts were two of the study authors (JC and MW) who independently reviewed each item in terms of applicability to ED staff. There was initial agreement on 51 (85%) items. Where there was disagreement, this was discussed between the two experts until consensus was achieved. Consensus occurred for all items. This process identified 28 items that were not relevant to the ED context, resulting in a 32-item measure to be subject to factor analyses.

Exploratory factor analysis

EFA and CFA should not be conducted on the same sample as this results in over-fitting and optimistic estimates of model fit within the CFA [33]. As such, the sample was randomly split into equal sized derivation and validation cohorts. EFA was conducted on the derivation cohort to undertake further item reduction and to identify the factor structure of the reduced scale. The number of factors to be retained was identified based on a number of strategies. These included 1) the number of factors with eigenvalues greater than one, 2) examination of the scree plot to identify the number of factors where eigenvalues start to level off [34], 3) parallel analysis, where the number of factors to be retained reflects the factors where eigenvalues are higher than those computed from a simulated random dataset [35], and 4) interpretability of the factor solution. The Kaiser-Meyer-Olkin (KMO) Test for sampling adequacy was conducted to determine the suitability of items for factor analysis. Items with a value ≥0.70 show moderate suitability, while values ≥0.80 show good suitability for factor analysis [36]. Values <0.70 were considered for exclusion in the current study. Further item reduction was based on factor loadings. Items that loaded highly (≥0.40) onto one component with low cross-loadings (≤0.20) on other factors were retained [34]. All other items were excluded, unless they were deemed to be an important coping strategy that was not captured by any retained items.

The derivation sample comprised 437 respondents, with a median age of 33 (IQR 27-44), and 78% female. There were 41 (9%) individuals with missing data on any of the JCS items, with the range of missing data for individual items being 1 (0.2% of respondents) to 11 missing responses (3% of respondents). There were no differences in median (or mean) JCS scores for those with and without missing data. As there were minimal missing data on any individual item, and few suitable methods for imputation of missing data in EFA [37], pairwise deletion of missing data was utilized for this analysis.

For peer review only - http://bmjopen.bmj.com/site/about/guidelines.xhtml

The KMO measure of the 32 JCS items revealed that 30 items were adequate for factor analysis, with the two items focusing on exercising and acceptance having low factorability. As there were remaining items within the JCS that assessed similar coping strategies to these 2 items, these were removed, leaving 30 items for exploratory factor analysis. Parallel analyses, and scree plots on the 30 items suggested that there were 3 to 6 factors underlying the items. Factor analyses with oblimin rotation were run using 3, 4, 5 and 6 factors. In each of these analyses, eight items had low loadings on all factors, or had low loadings with high cross loadings. These items focussed on worry, considering alternative methods for handing the situation, reframing the problem, getting mad, internalizing the problem, relaxation, distraction, and action planning. Again, these coping strategies were reflected in remaining JCS items and they were removed. The remaining 22-items fell on between 3 and 5-factors. However, the 4 and 5 factor solutions displayed factor splitting, with only a small number of items falling with low cross-loadings on the final factor. The 3-factor solution yielded interpretable factors. Based on the three-factor model, four additional items were removed (discussing the problem with significant others, objective perspective, blaming others, and minimizing the problem) for low loadings with high cross-loadings. All remaining items were retained as they were felt to be important for the scale. The KMO for these final 18 items was 0.8 while Bartlett's test of sphericity was $\chi^2 =$ 2,322, p<0.001. Both indicate adequate factorability. The final solution is provided in Table 2. The three factors were labeled problem-focussed coping (4 items), negative emotion-focussed coping (10-items), and positive emotion-focussed coping (4 items).

Table 2. Standardized factor loadings for factor structure outlined in exploratory factor analysis

Factors and Items†	Factor Loadings		
	NE	PE	PF
Negative Emotion-Focussed Coping			
3. Used smoking or medication for stress relief	**0.61**	0.01	0.06
9. Pessimistic thinking	**0.52**	-0.03	0.20
22. Spent time alone	**0.58**	0.15	-0.09
34. Drank	**0.46**	-0.03	0.09
46. Risky behaviour	**0.61**	0.04	0.05
48. Ignored problem	**0.58**	0.11	-0.25
51. Self-blame for problem	**0.51**	-0.12	0.16

For peer review only - http://bmjopen.bmj.com/site/about/guidelines.xhtml

53. Took stress-reducing medications	**0.61**	-0.10	0.20
56. Physical distancing	**0.80**	-0.06	-0.13
58. Wishful thinking	**0.49**	0.01	0.04
Positive Emotion-focussed Coping			
37. Hardiness attitude	0.14	**0.48**	0.07
39. Used humour	0.11	**0.49**	0.04
50. Optimistic thinking	-0.05	**0.82**	0.05
54. Refocus on good side	-0.02	**0.76**	-0.03
Problem-focussed coping			
15. Discussed problem with professional	-0.04	-0.07	**0.66**
27. Information seeking	0.07	0.13	**0.63**
42. Discussed problem with someone who has experienced the situation	0.00	0.13	**0.51**
45. Learned new skills	-0.04	0.26	**0.43**

†Items have been summarized. Complete items are from the JCS-A [16] © Anne Jalowiec and are available on request. Bolded items are primary factor loading. NE=Negative emotion-focussed coping; PE=Positive emotion-focussed coping; PF=Problem-focussed coping

Confirmatory Factor Analysis

This factor structure identified through EFA was validated using confirmatory factor analysis on the remaining cohort. The validation sample comprised 438 individuals with a median age of 36 (IQR=28-46). Within the validation cohort, there were 37 respondents (8%) who had missing data on one or more JCS items. Missing data were imputed using ten datasets that were developed using the remaining JCS items, age, gender and country as predictors. Polychoric correlations were again utilised and the diagonally weighted least squares estimator was used to estimate model parameters [38]. Robust corrections were used to adjust for any non-independence of errors associated with clustering across sites. However, ICCs were very low for all items (Median = 2%, IQR=1 to 5%), suggesting that there was minimal site level variance in JCS items.

A variety of goodness of fit measures were utilized in the current study to assess different aspects of model fit. These include absolute fit measures ($\chi^{2,}$ and the standardized root mean square residual [SRMR]), a parsimony correction index (the root mean square error of

For peer review only - http://bmjopen.bmj.com/site/about/guidelines.xhtml

approximation [RMSEA], and comparative fit indices (the comparative fit index [CFI] and the Tucker-Lewis index [TLI]) [25]. Support for the target model is obtained where SRMR is close to 0.08 or below, RMSEA values are close to 0.06 or below, and CFI and TLI values are close to 0.90 or greater [39].Fitting the three-factor solution to the validation sample provided a good fit to the data (χ^2= 323, p <0.01, CFI=0.93, TLI=0.92, SRMR=0.08, RMSEA=0.06) and all items loaded significantly onto their respective factors. Standardized loadings are provided in Table 3.

Table 3. Standardized factor loadings for theorised 3-factor model in confirmatory factor analysis

Factor and Items†	Factor Loadings		
	NE	PE	PF
Negative Emotion-Focussed Coping (NE)			
3. Used smoking or medications for stress relief	0.44		
9. Pessimistic thinking	0.38		
22. Spent time alone	0.63		
34. Drank	0.46		
46. Risky behaviour	0.56		
48. Ignored problem	0.64		
51. Self-blame for problem	0.63		
53. Took stress-reducing medications	0.53		
56. Physical distancing	0.77		
58. Wishful thinking	0.59		
Positive Emotion-focussed Coping (PE)			
37. Hardiness attitude		0.55	
39. Used humour		0.56	
50. Optimistic thinking		0.81	
54. Refocus on good side		0.83	
Problem-focussed coping (PF)			
15. Discussed problem with professional			0.46
27. Information seeking			0.44
42. Discussed problem with someone who has experienced the situation			0.59
45. Learned new skills			0.57

For peer review only - http://bmjopen.bmj.com/site/about/guidelines.xhtml

†Items have been summarized. Complete items are from the JCS-A [16] © Anne Jalowiec and are available on request. NE=Negative emotion-focussed coping; PE=Positive emotion-focussed coping; PF=Problem-focussed coping

To further explore the data, a number of additional (post hoc) models were fit to identify whether the factor structure was similar across job roles and across country. Given sample size limitations for some groups, these analyses were fit on the entire cohort rather than focusing only on the validation cohort. For each comparison, several invariance models were fit in the order specified by Wu and Estabrook [40] . Goodness of fit measures were compared across models requiring increasing invariance, including invariance of factor structure, thresholds, and loadings. For job role, the goodness of fit measures for all models were similar, indicating that both factor structure and factor loadings were similar for doctors and nurses (Supplementary Table 1). For country, goodness of fit measures were acceptable for all models. However, they were best for the factor structure model. This indicates that the factor structure was similar for Swedish and Australian respondents, but the strength of individual factor loadings were slightly different across countries. Factor loadings for models where loadings were allowed to vary are provided in Supplementary Tables 2 and 3) and show only minor differences in factor loadings across groups.

Descriptive Data and Criterion Validity

Descriptive data for the overall cohort and by country are reported in Table 4. Cronbach alphas were 0.77 for negative-emotion focussed coping, 0.68 for positive-emotion focussed coping, and 0.61 for problem-focussed coping. Alphas could not be improved through the removal of any individual item. Negative emotion-focussed coping strategies were used less often than positive emotion-focussed coping or problem-focussed coping strategies. The cohort from Sweden reported slightly less use of negative coping strategies and slightly more use of positive and problem-focussed strategies.

For peer review only - http://bmjopen.bmj.com/site/about/guidelines.xhtml

Table 4: Coping strategies by country

	Overall Cohort	Australia	Sweden	Difference (95% CI of difference)
Negative Emotion-Focussed Coping	1.0 (0.5)	1.1 (0.5)	0.8 (0.4)	-0.3 (-0.3 to 0.2)
Positive Emotion-Focussed Coping	2.1 (0.6)	2.1 (0.5)	2.3 (0.6)	0.2 (0.1 to 0.3)
Problem-focussed Coping	1.9 (0.6)	1.9 (0.6)	2.1 (0.5)	0.2 (0.1 to 0.3)

The scale ranges from 0 (never used) to 3 (often used). CI=confidence interval

Coefficients from the regression of coping strategies on WES-10 subscales are provided in Table 5. There was an association between the use of negative emotion-focussed coping strategies and poor perceptions of the work environment (lower opportunity for personal and professional growth, higher workload, higher conflict among staff members and higher nervousness or tension about going to work). The use of either problem-focussed coping strategies or positive emotion-focussed coping strategies had a weak association with perceptions that the workplace provided opportunity for personal and professional growth (self-realization).

Coefficients from the regression of coping strategies on ratings of stressors are provided in Table 5. Respondents reporting higher use of negative emotion-focussed coping strategies rated events to be more stressful. There was no association between ratings of stressors and either positive emotion- focussed coping or problem-focussed coping. One exception was that higher problem-focussed coping had a weak relationship with higher stress over inability to provide optimum care.

For peer review only - http://bmjopen.bmj.com/site/about/guidelines.xhtml

For peer review only

Table 5. Regression coefficients and 95% confidence intervals from coping strategies with WES-10 subscales and job stressors.

	Negative Emotion-Focussed Coping	Positive Emotion-Focussed Coping	Problem-Focussed Coping
WES-10 subscales			
Self-Realization	-0.3 (-0.4 to -0.2)	0.2 (0.1 to 0.2)	0.1 (0.1 to 0.2)
Workload	0.3 (0.2 to 0.3)	0 (0 to 0.1)	0.1 (0 to 0.2)
Conflict	0.5 (0.4 to 0.6)	0 (-0.1 to 0.1)	0.1 (0 to 0.2)
Nervousness	0.6 (0.5 to 0.7)	-0.1 (-0.2 to 0)	0 (-0.1 to 0.1)
ED SS			
Workplace violence	0.9 (0.4 to 1.4)	0.2 (-0.3 to 0.6)	0.2 (-0.2 to 0.7)
Heavy workload	1.5 (1.1 to 1.8)	-0.2 (-0.6 to 0.1)	0.2 (-0.2 to 0.5)
High acuity patients	1.4 (1.0 to 1.8)	-0.3 (-0.7 to 0.1)	0 (-0.4 to 0.4)
Inability to provide optimum care	1.5 (1.1 to 1.9)	0 (-0.3 to 0.4)	0.7 (0.3 to 1.1)
Environmental concerns	1.6 (1.1 to 2.0)	-0.1 (-0.5 to 0.3)	0.4 (0.0 to 0.8)

WES=work environment scale (10 items). ED SS = Emergency Department stressor scale. The coefficient represents the predicted change in WES-10 scores or ED stressor scales for each 1-point increase in coping strategies.

DISCUSSION

This study utilised data from a large sample of international ED staff to develop a refined JCS for future use by ED staff. The final scale incorporated 18 items measuring three styles of coping; problem-focussed coping, positive emotion-focussed coping, and negative emotion-focussed coping. It is short enough to encourage inclusion in future surveys exploring ED staff perceptions. Data exploration using this tool indicated that the use of negative coping strategies was associated with poor perceptions of the work environment and higher ratings of ED job stressors in both Australian and Swedish EDs. This modified scale is likely to be useful in identifying the types of coping strategies employed by ED staff and for use in evaluating interventions to reduce the use of maladaptive coping strategies.

For peer review only - http://bmjopen.bmj.com/site/about/guidelines.xhtml

The three categories of coping identified in the present study; problem-focussed coping, positive emotion-focussed coping, and negative emotion-focussed coping aligned with other theoretical literature. For example, the division of problem and emotion-focussed coping accords with Lazarus's theoretical model of coping [5]. This categorization also acknowledges that there are different styles of emotion-focussed coping, some potentially adaptive, and others potentially maladaptive [8]. By developing a shortened JCS that corresponds with theoretical and empirical research on coping, we have not only developed an ED based tool to explore coping strategies, but also a tool that is likely to be suitable for administration more broadly in the busy environments experienced by acute care health professionals. However, further validation would be required to identify broader utility of this scale.

Using the modified JCS enabled the team to establish an association between negative emotion-focused coping and poor perceptions of the working environment, as well as higher ratings of stress around listed events. This pattern of response is consistent with previous research [3, 9, 41] and accords with pragmatic perceptions of the development of burnout in ED staff. Maladaptive coping strategies have been consistently linked to poor perceptions of stress management [41], poor psychological health [3] and burnout [9]. In contrast, problem-focused and positive emotion-focused strategies were largely unrelated to perceptions of the working environment and perceptions of stress around specific events. This finding may appear to differ from previous studies finding that problem-focused strategies in particular have beneficial outcomes, such as lower burnout and distress [3, 9]. However, this study focussed on ratings of stressors, rather than the outcomes of stress (e.g., psychological distress and burnout). Additional research relating these scales to health outcomes would be required to clarify this relationship. Further, the broader literature does note that problem- and emotion-focused strategies may not be classed as inherently good or bad [5]. Instead, their effectiveness depends on the situation [8]. In situations where stressors are controllable (e.g., taking an exam), problem-focused coping is useful [42]. Conversely, when dealing with stressors such as a major loss, it may be adaptive initially to engage in some palliative coping [43]. As such, these strategies may not show clear relationships with broad outcome variables.

The strengths of this study include the generation of a validated revised JCS using a large multidisciplinary international sample. The limitations include the lack of availability of longitudinal data, meaning that test-retest reliability was unable to be determined. Test-retest reliability is necessary to ensure that the scale is reliable and stable in assessing coping strategies across

For peer review only - http://bmjopen.bmj.com/site/about/guidelines.xhtml

time. The response rate was reasonable for a survey design. However, it is unknown whether the respondents were representative of the broader population. No data were available on the specific response rate for nurses and physicians. Nurses made up the majority of the sample at a number of sites and it is unclear whether this accurately reflects the workforce, or whether there was low response rate by physicians in those sites. No data were available on burnout or employee mental health and so limited assessment of the outcomes of coping could be conducted. The JCS assesses coping using a 4-item Likert scale. Research has shown that the reliability, validity and discriminating power of four-point scale is relatively poor, with the optimum number of categories being between 7 to 10 [44]. Future research may benefit from incorporating a larger number of response categories.

The ED can be a particularly stressful workplace. Stressors can result in increased sick leave and staff burnout, poor staff recruitment and retention, decreased staff morale, and decreased job satisfaction. Understanding ways in which ED staff cope with stressors that pertain to their work is important. We have developed a revised JCS to assess the coping strategies utilised by ED staff. This shortened version of the JCS incorporates ten maladaptive emotion focussed strategies incorporating aspects such as risky behavior, drinking, stress-reducing medications and ignoring the problem. It also includes four positive emotion focussed strategies around refocusing, being optimistic and using humor. Finally, the scale includes four problem-focussed coping strategies including information seeking and learning new skills. This scale may be used to evaluate the outcome of interventions that seek to promote positive coping strategies.

FUNDING

This study was supported by an Emergency Medicine Foundation grant (EMSS-410R22-2014)

CONFLICTS OF INTEREST

The authors declare no competing interests

AUTHOR STATEMENT

JG, MW, AJ, EC, DW, OT, LA and JC conceived and designed the study. MW, AJ, EC, DW, OT, LA and JC. were involved in acquisition of data. JG analysed the data. MW, AJ, EC, DW, and JC assisted with interpretation of the data. JG, MW and JC drafted the work and all authors revised the manuscript critically for important intellectual content.

For peer review only - http://bmjopen.bmj.com/site/about/guidelines.xhtml

DATA STATEMENT

There are no additional data available

REFERENCES

1 Cronin G, Cronin C. Why does A&E attract newly qualified registered nurses? Accid Emerg Nurs 2006;14:71-7.
2 Rozo JA, Olson DM, Thu H, et al. Situational factors associated with burnout among emergency department nurses. Workplace Health Saf 2017;65(6):262-5.
3 Yates PJ, Benson EV, Harris A, et al. An investigation of factors supporting the psychological health of staff in a UK emergency department. Emerg Med J 2012;29:533-5.
4 Sawatzky JV, Enns CL. Exploring the key predictors of retention in emergency nurses. J Nurs Manag 2012;20:696-707.
5 Lazarus RS, Folkman S. Stress, appraisal, and coping. New York: Springer; 1984.
6 Lazarus RS. From psychological stress to the emotions: A history of changing outlooks. Ann Rev Psychol 1993;44:1-21.
7 Folkman S, Lazarus RS. Coping as a mediator of emotion. J Pers Soc Psycho 1988;54(3):466-75.
8 Folkman S, Moskowitz JT. Coping: Pitfalls and Promise. Ann Rev Psychol 2004;2004(55):745-74.
9 Howlett M, Doody K, Murray J, et al. Burnout in emergency department healthcare professionals is associated with coping style: a cross-sectional survey. Emerg Med J 2015;32:722-7.
10 Lu D-M, Sun N, Hong S, et al. Occupational stress and coping strategies among emergency department nurses of china. Arch Psychiatr Nurs 2015;29:208-12.
11 Zavotsky KE, Chan G. Exploring the relationshp among moral distress, coping, the practice environment in Emergency Department nurses. Advanced Emergency Nursing Journal 2016;38:133-46.
12 Jeong I-Y, Kim J-S. The relationship between intention to leave the hospital and coping methods of emergency nurses after workplace violence. Journal of Clinical Nursing 2018;27:1692-701.
13 Donoghue KJ. Measuring coping: Evaluating the psychometric properties of the COPE: Edith Cowan Univeristy; 2004.
14 Kato T. Frequently used coping scales: A meta-analysis. Stress and Health 2013;31:315-23.
15 Lyne K, Roger D. A psychometric re-assessment of the COPE questionnaire. Personality and Indvidual Differences 2000;29(2):321-35.
16 Jalowiec A. The Jalowiec Coping Scale. In: Strickland O, editor. Measurement of Nursing Outcomes New York: Springer; 2003. p. 71–97.
17 Jalowiec A. Jalowiec Coping Scale: Overview of JCS by Dr. Anne Jalowiec 2001. http://www.allcare.org/CancerPain-and-SymptomManagement/wellbe/wbm4/Jalowiec%20coping%20scale%20overview.pdf. (accessed
18 Ulvik B, Johnsen TB, Nygard O, et al. Factor structure of the revised Jalowiec Coping Scale in patients admitted for elective coronary angiography. Scand J Caring Sci 2008;22(4):596-607.
19 Røssberg JI, Eiring Ø, Friis S. Work environment and job satisfaction: A psychometric evaluation of the Working Environment Scale-10. Soc Psychiatry Psychiatr Epidemiol 2004;39:576-80.
20 Crilly J, Greenslade JH, Lincoln C, et al. Measuring the impact of the working environment on emergency department nurses: A cross-sectional pilot study. Int Emerg Nurs 2017;31:9-14.
21 Ross-Adjie GM, Leslie G, Gillman L. Occupational stress in the ED: What matters to nurses? Australas Emerg Nurs J 2007;10:117-23.
22 R Core Team. R: A language and environment for statistical computing. Vienna, Austria: R Foundation for Statistical Computing; 2018.

For peer review only - http://bmjopen.bmj.com/site/about/guidelines.xhtml

23 Revelle W. psych: Procedures for Psychological, Psychometric, and Personality Research. Evanston, Illinois: Northwestern Univeristy; 2018.
24 Lee C-T, Zhang G, Edwards MC. Exploratory least squares estimation of parameters in exploratory factor analysis with ordinal data. Multivariate Behav Res 2012;47(2):314-39.
25 Brown TA. Confirmatory factor analysis for applied research, second edition. New York: Guilford Publications; 2015.
26 Rosseel Y. lavaan: An R package for structural equation modeling. J Stat Softw 2012;48(2):1-36.
27 Borneman MJ. Criterion Validity. In: Salkind NJ, editor. Encyclopedia of Research Design: SAGE; 2010.
28 Elder EG, Johnston A, Wallis M, et al. Emergency clinician perceptions of occupational stressors and coping strategies: a multi-site study. Int Emerg Nurs 2019.
29 Xu H, Johnston A, Greenslade JH, et al. Stressors and coping strategies of emergency department nurses and doctors: A cross-sectional study. Australasian Emergency Care 2018.
30 Autralian Institute of Health and Welfare. Nursing and midwifery workforce 2015. Cat. no. WEB 141. Canberra: AIHW; 2016.
31 Lindqvist R, Alenius LS, Runesdotter S, et al. Organization of nursing care in three Nordic countries: relationships between nurses' workload, level of involvement in direct patient care, job satisfaction, and intention to leave. BMC Nursing 2014;13:27.
32 Australian Institute of Health and Welare. Medical practitioners workforce 2015. Cat. no. WEB 140. . Canberra, Australia: AIHW; 2016.
33 Fokkema M, Greiff S. How performing PCA and CFA on the same data equals trouble: overfitting in the assessment of internal structre and some editorial thoughts on it. Eur J Psychol Assess 2017;33(6):399-402.
34 Matsunaga M. How to Factor-Analyze Your Data Right: Do's, Don'ts, and How-To's. Int J Psychol Res 2010;3(1):97-110.
35 Horn JL. A rationale and test for the number of factors in factor analysis. Psychometrika 1965;30(2):1790185.
36 Kaiser H. An index of factor simplicity. Psychometrika 1974;39:31-6.
37 Çokluk O, Kayri M. The Effects of Methods of Imputation for Missing Values on the Validity and Reliability of Scales. Educational Sciences: Theory and Practice 2011;11(1):303-9.
38 Li C-H. The performance of ML, DWLS, and ULS estimation with robust corrections in structural equation models with ordinal variables. Psychol Methods 2016;21(3):369-87.
39 Hu L-t, Bentler PM. Cutoff criteria for fit indexes in covariance structure analysis: Conventional criteria versus new alternatives. Struct Equ Modeling 1999;6:1-55.
40 Wu H, Estabrook R. Identification of confirmatory factor analysis models of different levels of invariance for ordered categorical outcomes. Psychometrika 2016;81:1015-45.
41 Holton MK, Barry AE, Chaney D. Employee stress management: An examination of adaptive and maladaptive coping strategies on employee health. Work 2016;53:299-305.
42 Folkman S, Lazarus RS. If it changes it must be a process: study of emotion and coping during three stages of a college examination. J Pers Soc Psycho 1985;48:150-70.
43 Stroebe MS, Schut H, editors. Meaning making in the dual process model of coping with bereavement. Washington, DC: American Psychological Association; 2001.
44 Preston CC, Colman AM. Optimal number of response categories in rating scales: reliability, validity, discriminating power, and respondent preferences. Acta Psychol 2000;104:1-15.

.

For peer review only - http://bmjopen.bmj.com/site/about/guidelines.xhtml

For peer review only

For peer review only - http://bmjopen.bmj.com/site/about/guidelines.xhtml

Supplementary Table 1. Goodness of fit for models comparing job role and country

Fixed parameters	χ^2 (df)	CFI	TLI	RMSEA	SRMR
Job Role					
Factor structure (configural model)	636 (264)	0.93	0.91	0.06	0.08
Threshold	700 (297)	0.92	0.92	0.06	0.08
Loadings	725 (312)	0.92	0.92	0.06	0.08
Country					
Factor structure (configural model)	586 (264)	0.94	0.93	0.05	0.08
Threshold	833 (297)	0.90	0.89	0.06	0.08
Loadings	927 (312)	0.88	0.89	0.07	0.08

For peer review only

Supplementary Table 2. Standardized factor loadings for factor structure outlined in confirmatory factor analysis for nurses and physicians

Factors and Items†	Factor Loading Nurses			Factor Loadings Physicians		
	NE	PE	PF	NE	PE	PF
Negative Emotion-Focussed Coping						
3. Used smoking or medication for stress relief	**0.50**			**0.57**		
9. Pessimistic thinking	**0.48**			**0.38**		
22. Spent time alone	**0.60**			**0.61**		
34. Drank	**0.48**			**0.36**		
46. Risky behaviour	**0.58**			**0.56**		
48. Ignored problem	**0.58**			**0.65**		
51. Self-blame for problem	**0.59**			**0.58**		
53. Took stress-reducing medications	**0.58**			**0.52**		
56. Physical distancing	**0.76**			**0.82**		
58. Wishful thinking	**0.55**			**0.55**		
Positive Emotion-focussed Coping						
37. Hardiness attitude		**0.52**			**0.51**	
39. Used humour		**0.52**			**0.55**	
50. Optimistic thinking		**0.85**			**0.71**	
54. Refocus on good side		**0.77**			**0.82**	
Problem-focussed coping						

For peer review only

15. Discussed problem with professional	**0.47**	**0.48**
27. Information seeking	**0.58**	**0.61**
42. Discussed problem with someone who has experienced the situation	**0.57**	**0.59**
45. Learned new skills	**0.63**	**0.55**

For peer review only

Supplementary Table 3. Standardized factor loadings for factor structure outlined in confirmatory factor analysis for Australia and Sweden

Factors and Items†	Factor Loading Sweden			Factor Loadings Australia		
	NE	PE	PF	NE	PE	PF
Negative Emotion-Focussed Coping						
3. Used smoking or medication for stress relief	**0.62**			**0.44**		
9. Pessimistic thinking	**0.45**			**0.46**		
22. Spent time alone	**0.54**			**0.60**		
34. Drank	**0.47**			**0.39**		
46. Risky behaviour	**0.55**			**0.57**		
48. Ignored problem	**0.42**			**0.65**		
51. Self-blame for problem	**0.53**			**0.60**		
53. Took stress-reducing medications	**0.67**			**0.54**		
56. Physical distancing	**0.63**			**0.77**		
58. Wishful thinking	**0.53**			**0.63**		
Positive Emotion-focussed Coping						
37. Hardiness attitude		**0.48**			**0.53**	
39. Used humour		**0.56**			**0.62**	
50. Optimistic thinking		**0.91**			**0.76**	
54. Refocus on good side		**0.78**			**0.77**	
Problem-focussed coping						

For peer review only

15. Discussed problem with professional	**0.64**	**0.43**
27. Information seeking	**0.48**	**0.62**
42. Discussed problem with someone who has experienced the situation	**0.81**	**0.54**
45. Learned new skills	**0.58**	**0.60**

For peer review only

STROBE 2007 (v4) Statement—Checklist of items that should be included in reports of *cross-sectional studies*

Section/Topic	Item #	Recommendation	Reported on page #
**Title and abstract**	1	(*a*) Indicate the study's design with a commonly used term in the title or the abstract	1
		(*b*) Provide in the abstract an informative and balanced summary of what was done and what was found	3
**Introduction**			
Background/rationale	2	Explain the scientific background and rationale for the investigation being reported	5
Objectives	3	State specific objectives, including any prespecified hypotheses	6
**Methods**			
Study design	4	Present key elements of study design early in the paper	6
Setting	5	Describe the setting, locations, and relevant dates, including periods of recruitment, exposure, follow-up, and data collection	6
Participants	6	(*a*) Give the eligibility criteria, and the sources and methods of selection of participants	6
Variables	7	Clearly define all outcomes, exposures, predictors, potential confounders, and effect modifiers. Give diagnostic criteria, if applicable	6-7
Data sources/ measurement	8*	For each variable of interest, give sources of data and details of methods of assessment (measurement). Describe comparability of assessment methods if there is more than one group	6-7
Bias	9	Describe any efforts to address potential sources of bias	N/A
Study size	10	Explain how the study size was arrived at	6
Quantitative variables	11	Explain how quantitative variables were handled in the analyses. If applicable, describe which groupings were chosen and why	7-9
Statistical methods	12	(*a*) Describe all statistical methods, including those used to control for confounding	7-9
		(*b*) Describe any methods used to examine subgroups and interactions	N/A
		(*c*) Explain how missing data were addressed	8
		(*d*) If applicable, describe analytical methods taking account of sampling strategy	N/A
		(*e*) Describe any sensitivity analyses	N/A
**Results**			

For peer review only

Participants	13*	(a) Report numbers of individuals at each stage of study—eg numbers potentially eligible, examined for eligibility, confirmed eligible, included in the study, completing follow-up, and analysed	6
		(b) Give reasons for non-participation at each stage	N/A
		(c) Consider use of a flow diagram	N/A
Descriptive data	14*	(a) Give characteristics of study participants (eg demographic, clinical, social) and information on exposures and potential confounders	10
		(b) Indicate number of participants with missing data for each variable of interest	8
Outcome data	15*	Report numbers of outcome events or summary measures	N/A
Main results	16	(*a*) Give unadjusted estimates and, if applicable, confounder-adjusted estimates and their precision (eg, 95% confidence interval). Make clear which confounders were adjusted for and why they were included	11-14
		(*b*) Report category boundaries when continuous variables were categorized	N/A
		(*c*) If relevant, consider translating estimates of relative risk into absolute risk for a meaningful time period	N/A
Other analyses	17	Report other analyses done—eg analyses of subgroups and interactions, and sensitivity analyses	N/A
**Discussion**			
Key results	18	Summarise key results with reference to study objectives	15-16
Limitations	19	Discuss limitations of the study, taking into account sources of potential bias or imprecision. Discuss both direction and magnitude of any potential bias	16
Interpretation	20	Give a cautious overall interpretation of results considering objectives, limitations, multiplicity of analyses, results from similar studies, and other relevant evidence	17
Generalisability	21	Discuss the generalisability (external validity) of the study results	16
**Other information**			
Funding	22	Give the source of funding and the role of the funders for the present study and, if applicable, for the original study on which the present article is based	18

*Give information separately for cases and controls in case-control studies and, if applicable, for exposed and unexposed groups in cohort and cross-sectional studies.

Note: An Explanation and Elaboration article discusses each checklist item and gives methodological background and published examples of transparent reporting. The STROBE checklist is best used in conjunction with this article (freely available on the Web sites of PLoS Medicine at http://www.plosmedicine.org/, Annals of Internal Medicine at http://www.annals.org/, and Epidemiology at http://www.epidem.com/). Information on the STROBE Initiative is available at www.strobe-statement.org.

For peer review only
